# Rise in complications of acute otitis media during and after the COVID-19 pandemic

**DOI:** 10.1007/s00405-024-08647-4

**Published:** 2024-05-06

**Authors:** Hannes Hollborn, Christoph Lachmann, Daniel Strüder, Sara M. van Bonn, Robert Mlynski, Sebastian P. Schraven

**Affiliations:** 1 Department of Otorhinolaryngology, Head and Neck Surgery “Otto Körner”, Rostock University Medical Center, Doberaner Straße 137-139, 18057 Rostock, Germany; 2 Department of Otorhinolaryngology, Head and Neck Surgery, Aachen University Medical Center, Pauwelsstraße 30, 52074 Aachen, Germany

**Keywords:** Mastoidectomy, Acute mastoiditis, Acute otitis media, COVID-19, Nonpharmaceutical intervention

## Abstract

**Purpose:**

After the lifting of nonpharmaceutical interventions (NPIs) during the COVID-19 pandemic, clinical observation showed an increase in complications of acute otitis, followed by a rise in the number of mastoidectomies performed. The aim of this study was to record the number of mastoidectomies performed before, during and after the COVID-19 pandemic as an indicator for complications of acute otitis media.

**Methods:**

Data were collected from a tertiary hospital in a university setting, as well as from four major public health insurance companies in Germany. The data of 24,824,763 German citizens during a period from 2014 until 2023 were analyzed.

**Results:**

According to the data, during the COVID-19 pandemic, the number of mastoidectomies performed dropped by 54% for children aged 0–6 and by 62% for children aged 7–18. For adults, there were 30% fewer mastoidectomies performed between 2020 and 2022. After the lifting of most NPI’s in the season from July 2022 to June 2023, there was a sharp increase in the number of mastoidectomies performed on patients of all ages.

**Conclusions:**

During the COVID-19 pandemic, a decrease in the number of mastoidectomies performed was seen, suggesting a lower incidence of complicated acute otitis, most likely linked to the general decrease of upper airway infections due to NPI’s. In contrast, a sharp increase in the incidence of complicated otitis occurred after the hygiene measures were lifted. The current development causes a more frequent performance of mastoidectomies, thus entailing a change in the challenges for everyday clinical practice.

## Introduction

Acute otitis media is one of the most common infectious diseases in children and, therefore, has a major economic impact [1]. The disease is one of the main reasons for consultations of pediatric general practitioners and ENT physicians and antibiotic prescriptions in Europe [2].

Acute otitis media is a bacterial or mixed viral and bacterial infection, but is usually preceded by a viral upper respiratory tract infection [3].

**Fig. 1:**
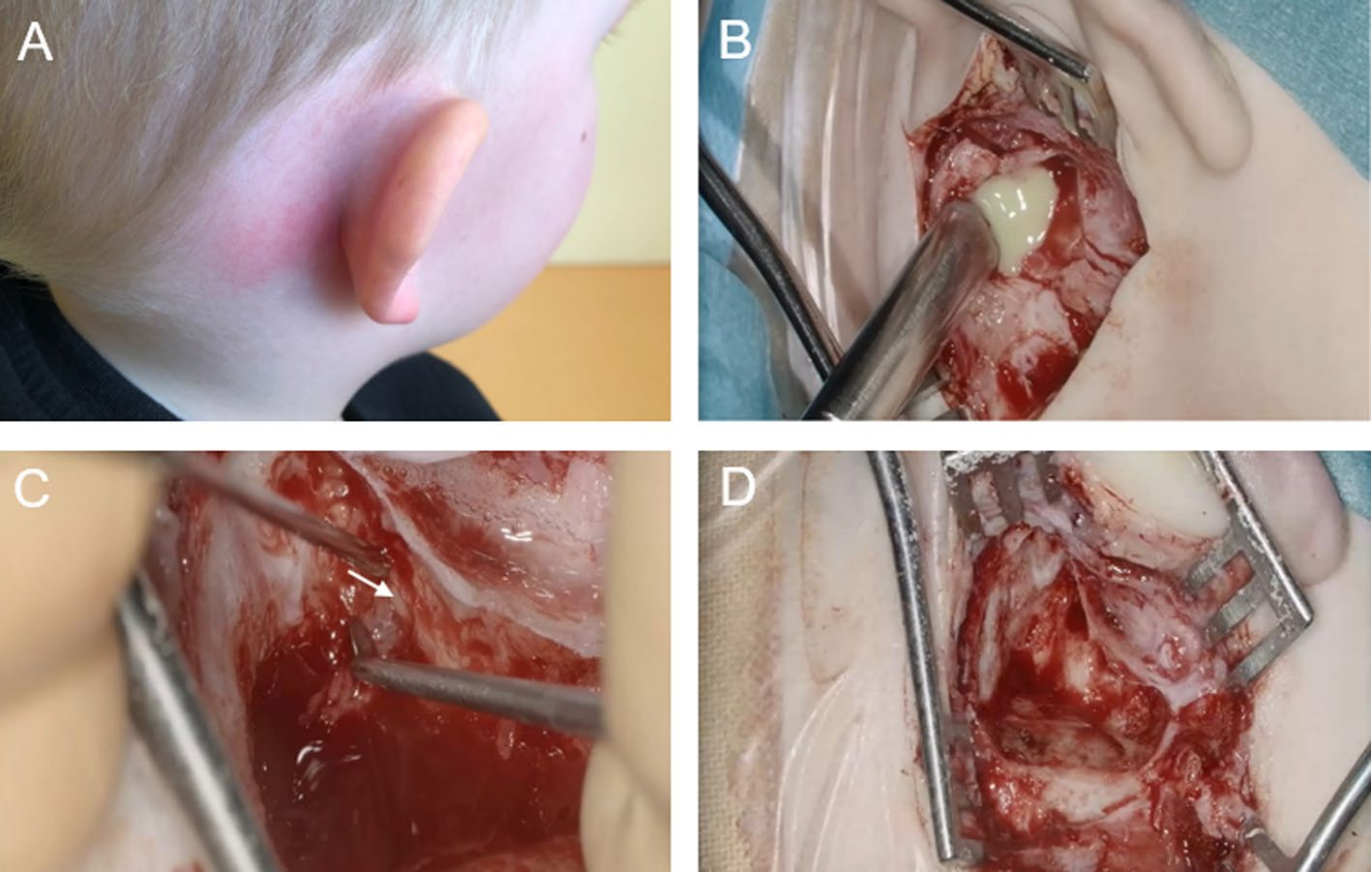
A 3-year-old boy presenting with typical clinical manifestations of an acute mastoiditis, including fever, otalgia, otapostasis and retroauricular swelling (**A**). Mastoidectomy (right ear) performed on a 3-year-old boy. **B** Showing the initial drainage of the subperiostal abscess. After identification of the dura mater the antrotomy (short crus of the incus indicated by the arrow) is performed (**B**). **C** Showing the situs after the finished mastoidectomy

In childhood, acute mastoiditis is one of the most common complications (Fig. 1). Due to the complex anatomy of the lateral skull base, there is a risk of spread of inflammation with life-threatening consequences, such as sinus vein thrombosis, meningitis, epi- or subdural abscess, and affection of the facial nerve or hearing loss as well as vestibular dysfunction. Rapid treatment is, therefore, essential. Mandatory intravenous antibiotic therapy and a tympanic drainage, in the case of a closed tympanic membrane are followed by a mastoidectomy for relief and, most commonly combined with adenotomy [4]. In childhood, antibiotic therapy and tympanic drainage combined with retroauricular puncture may be sufficient therapy, without the need for mastoidectomy [5]

Although the incidence of acute mastoiditis and the number of mastoidectomies remained stable in Germany during the 2010s, a significant reduction in complicated otitis was observed following the implementation of contact restrictions at the onset of the COVID-19 pandemic [6]. This decrease was associated with a significant reduction in upper respiratory tract infections, which occurred after the introduction of mandatory masks in Germany and in other countries, such as Korea in the spring of 2020 [7, 8]. Furthermore, elective surgical procedures, such as grommets or adenoid- and tonsillectomies were significantly reduced [9]. After the lifting of nonpharmaceutical interventions (NPIs—including, among others compulsory wearing of masks in public spaces, social distancing or curfews) in the winter season 2022/2023, an increased incidence of upper respiratory tract infections was observed [10]. Some of these infections were associated with severe courses of the disease. However, it has not yet been studied, whether there was also an increase in complicated acute otitis media. Therefore, the aim of this study is to analyze the incidence of complicated acute otitis media that led to a mastoidectomy. In this epidemiological retrospective analysis, in collaboration with major German health insurance companies), the number of mastoidectomies linked to complications of acute otitis media during the years preluding and during the COVID-19 pandemic was collected. This could be considered an indicator of complicated acute otitis media.

## Materials and methods

This is a retrospective analysis of the incidence of complicated otitis media, measured by the number of mastoidectomies performed for acute otitis media. The data were obtained from a tertiary university hospital in Germany and four major German health insurance companies. For the years 2014 to 2017, we collected the data from around 13,870,000 individuals per year. From 2018 to 2023, we received the data from approximately 24,820,000 individuals per year. In total, this corresponds to approximately 27% of the German population.

**Fig. 2:**
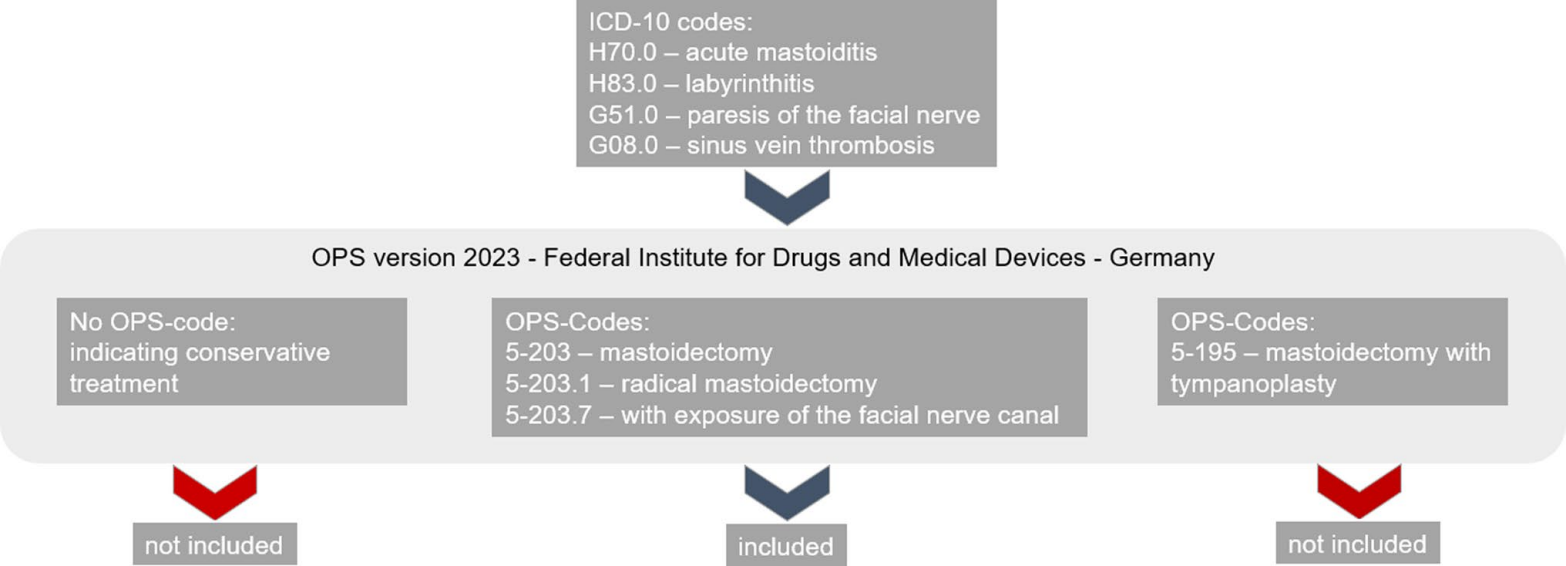
An overview of the selection of epidemiologic data. Patients who were hospitalized due to a diagnosis of acute mastoiditis were selected. Subsequently, all patients who received a mastoidectomy were considered anonymously. Patients treated conservatively or patients with a mastoidectomy together with a tympanoplasty were not considered

This study included patients of all ages who underwent mastoidectomy due to acute mastoiditis, or complications of acute otitis media, between January 1st, 2014 and June 30th, 2023. These patients were identified via the surgery archive, as shown in Fig. 2. Complications included labyrinthitis, facial nerve paresis, sinus vein thrombosis, intracranial abscesses, and sepsis. The right and left ear were considered separately. Patients with incomplete records or those who had undergone mastoidectomies for chronic conditions, such as cholesteatoma, or due to skull base surgery were excluded.

The data query was based on the international ICD-10 catalog (H70.0—acute mastoiditis) and the operation and procedure code (5-203.0—mastoidectomy).

To account for the seasonal accumulation of upper respiratory tract infections and the associated otitis in winter, the observation period was from July 1st to June 30th of the following year. This was designated in each case as the 2020/2021 season, etc. Patients were categorized into age groups 0–7 years, 8–18 years and 19–99 years. In addition, to preserve patient anonymity, the exact age at the time of surgery was not collected.

The study was approved by the university’s local Ethics Committee (A 2023-0159).

### Statistical analysis

Statistical analysis was conducted using SPSS Statistics software Version 22 (IBM Corp., Armonk, NY, USA). A Shapiro–Wilk test was performed to rule out a normal distribution of the collected data. The Kruskal–Wallis test was then used for statistical analysis of the data without normal distribution, while a variance analysis was used for data with normal distribution.

## Results

### Local study site

The local study site was a tertiary hospital in a university medical center in Germany. Patient data from 2015 to 2023 were available and analyzed.

**Fig. 3:**
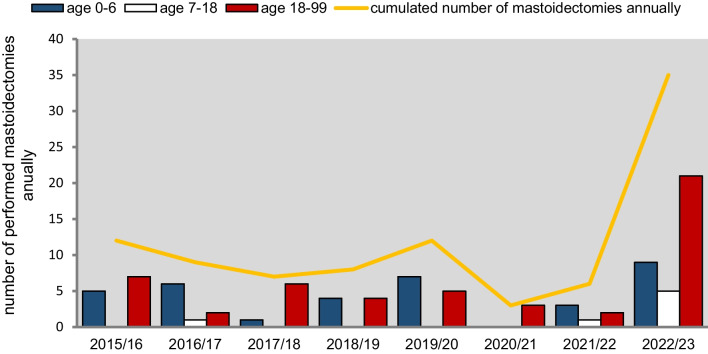
Change of the number of mastoidectomies over the years performed at the local study site, indicating a rise in complicated acute otitis media after lifting of the non-pharmaceutical interventions

Table 1 shows the number of mastoidectomies performed in each age group at the local study site between 2015 and 2023. In the years before the COVID-19 pandemic, a mean of 4.6 (± 2.1) mastoidectomies were performed annually in children 0–6 years of age, while school-age children underwent 0.2 (± 0.4) mastoidectomies, and adults underwent 4.8 (± 1.7) mastoidectomies. During contact restrictions, hygiene measures and the reduction of medical capacity under the COVID-19 pandemic, mastoidectomies decreased to a mean of 1.5 (± 1.5) per year in young children, 0.5 (± 0.5) in school-aged children, while 2.5 (± 0.5) were performed on adults annually. Notably, during the first season of the pandemic (2020/2021), when the most restrictive measures were in place, no mastoidectomies were performed on young children or school-aged children. Following the lifting of contact restrictions, mask mandates and release of medical capacity restrictions in the 2022/2023 season, there was a sharp increase in mastoidectomies across all age groups. For instance, 9 mastoidectomies were performed on children aged 0–6 years, 5 on school-aged children, and 21 on adults (Fig. 3). ﻿﻿Statistical significances could not be detected, due to the small sample size﻿﻿.

**Table 1:** Number of mastoidectomies performed for each age group during the seasons (01.07–31.06) from 2015 to 2023 at the local study site

	Age 0–6 years	Age 7–18 years	Age 19 and older
2015/16	5	0	7
2016/17	6	1	2
2017/18	1	0	6
2018/19	4	0	4
2019/20	7	0	5
2020/21	0	0	3
2021/22	3	1	2
2022/23	9	5	21

### Data from the health insurance companies

**Fig. 4:**
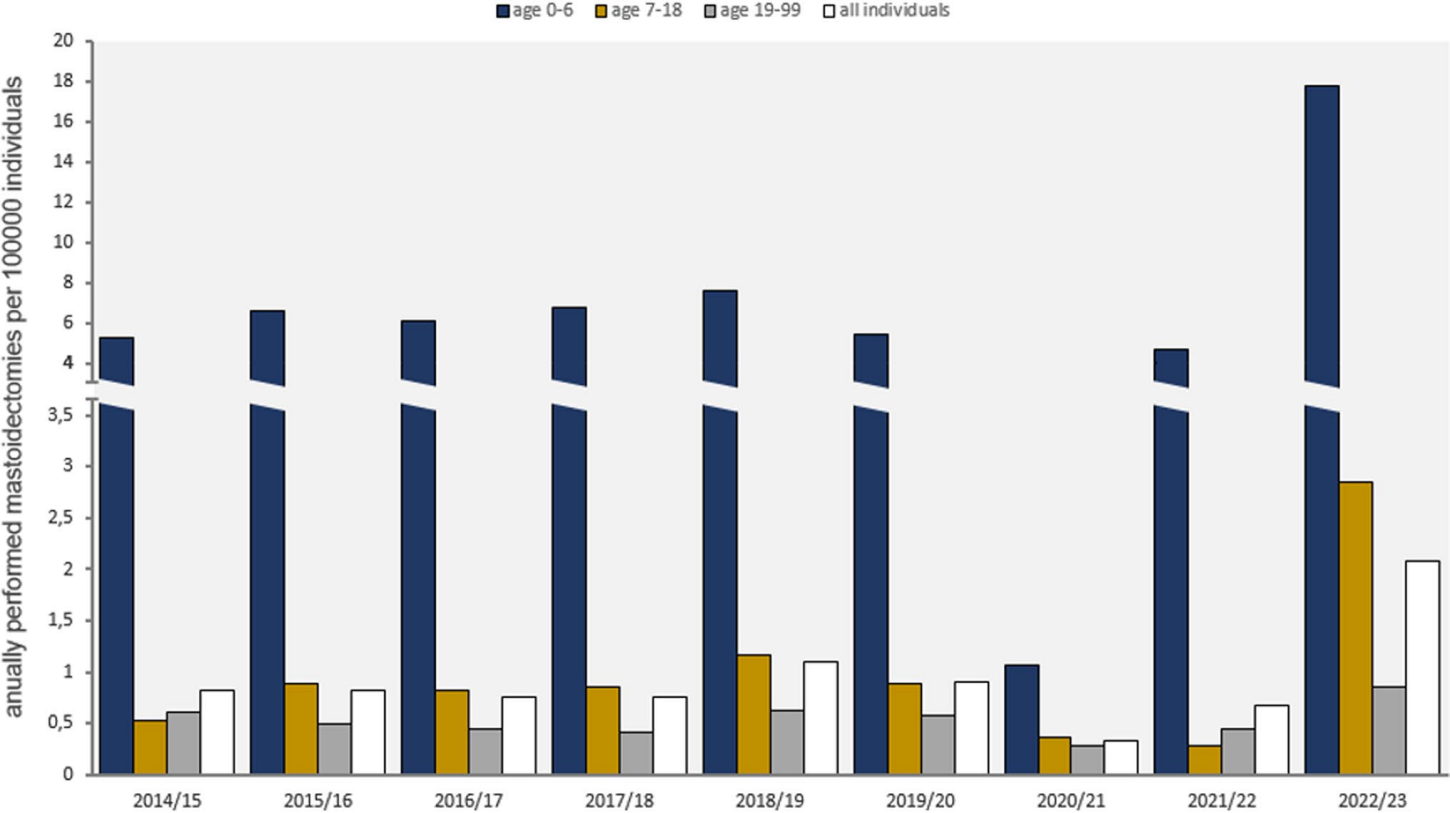
Change in the number of mastoidectomies per 100,000 individuals annually. Showing the impact of NPIs during the seasons 2020/2021 and 2021/2022, as well as the prominent increase in mastoidectomies after lifting of the NPIs

The data from the health insurance companies were received anonymously. Mastoidectomies performed due to acute otitis media with complications, or cases of acute mastoiditis for different age groups and seasons were analyzed. To calculate the seasonal incidence, the number of insured patients in each age group was used at the cutoff date (turn of the year). In total, we analyzed data from 24,824,763 insured individuals in Germany, including 1.5 million children aged 0–6 years, 2.5 million school-age children, and 20.7 million adults (Fig. 4).

To ensure comparability between data sets, the number of mastoidectomies performed annually was rounded to incidences per 100,000 inhabitants in the relevant age group.

#### Children aged 0–6 years

Before the COVID-19 pandemic, an average of 6.3 (± 0.79) mastoidectomies per 100,000 children aged 0–6 years were performed annually.

During the first year of the pandemic, under the NPIs, the number decreased to 1.1 mastoidectomies per 100,000 preschool-aged children. In the second year of the pandemic, the incidence was still slightly below the 2015–2019 mean at 4.7 mastoidectomies.

Following the removal of most NPIs, there was a sudden increase in mastoidectomies to 17.8 per 100,000 children aged 0–6 years during the 2022/2023 season.

#### Children and adolescents 7–18 years of age

School-aged children also experienced a decrease in mastoidectomies per 100,000 children during the COVID-19 pandemic, from a mean of 0.86 (± 0.19) before the pandemic to a mean of 0.33 (± 0.05) during the pandemic years.

In addition, there was an increase in mastoidectomies performed, to 2.86 per 100,000 children aged 7–18 years in the first season after most hygiene measures were lifted.

#### Adults 19 years and older

In the years before the pandemic, there were 0.53 (± 0.08) mastoidectomies per 100,000 inhabitants aged 19 years and older. During the pandemic years, 0.37 (± 0.08) mastoidectomies were performed. However, in the 2022/23 season, the number of procedures performed on adults more than doubled to 0.86 per 100,000 population, which is 1.6 times higher than in the pre-pandemic years.

#### General population

Across all age groups, the number of mastoidectomies performed annually per 100,000 individuals decreased from 0.86 (± 0.12) in the pre-pandemic years, to 0.51 (± 0.17) mastoidectomies per 100,000 individuals during the COVID-19 pandemic. After the lifting of most of the NPIs, the number of annual mastoidectomies per 100,000 individuals increased to 2.1. This represents an increase by 2.4 compared to pre-pandemic years and by 4.1 compared to the pandemic period.

#### Statistical analysis

A normal distribution of the collected data was ruled out with the Shapiro–Wilk test for the data for children aged 0–6 and 7–18, as well as for the general population. No statistically significant changes were detected in the number of annually performed mastoidectomies.

## Discussion

This nationwide epidemiological study, observed a decrease in complicated acute otitis requiring mastoidectomy was observed between 2020 and 2022 during the COVID-19 pandemic. In contrast, after the lifting of NPIs data shows a sharp rise in mastoidectomies performed, indicating a higher incidence of complicated acute otitis media. This change was particularly noticeable in children up to the age of 18.

These observations are consistent with epidemiological data from different countries. Initially, there were reports of a decrease in upper respiratory tract infectious diseases during the hygiene measures of the COVID-19 pandemic [11, 12]. For instance, Hullegie et al. also explicitly reported a decrease in acute otitis media disease in children in the Netherlands [13].

Acute otitis media is often a mixed infection that ascends from an acute respiratory infection. Therefore, a decrease in complicated acute otitis associated with a falling incidence of upper respiratory tract infections can be expected. The introduction of mandatory mask use [14] and other NPIs have both been shown to play an important role in this [15].

Additional factors that may have contributed to a decrease in mastoidectomies need to be discussed. During the peak of the COVID-19 pandemic, the general public may have been more sensitive to signs of complicated infections, which could have resulted in more rapid therapy and less frequent need for surgical intervention. In addition, clinicians may have opted for more conservative treatment with indicating fewer mastoidectomies to avoid surgeries in general. It is also possible that some patients avoided large hospitals due to fear of infection, which could have contributed to these trends. Cases that otherwise would have been borderline indications for mastoidectomy, remained under a conservative treatment regimen.

The increase in the number of mastoidectomies performed in the observed population is striking. Several factors could contribute to this trend. Firstly, there may have been a buildup of “immune debt” due to reduced exposure to pathogens during the hygiene measures of the COVID-19 pandemic. This effect is expected to be particularly strong in infants who were born after the outbreak of the COVID-19 pandemic, who had lower exposure to respiratory tract infection pathogens [16]. After the discontinuation of NPIs, there is a postulated rebound effect for the general population [17]. This is associated with a general increase in upper and lower respiratory tract infections, as exemplified by the respiratory syncytial virus. Many countries, including Canada [18], Turkey [19], and Germany [20], have reported an increase in complicated respiratory syncytial virus infections during the 2022/2023 season. Moreover, there might have been an emergence of new aggressive bacterial clusters, causing an increase in complicated airway infections. An example of this was the new cluster of streptococcus type A found in France in 2022 [21]

Another possible reason for the rise in acute mastoiditis in young children could be the decrease in adenotomies performed during the COVID-19 pandemic [9]. Due to the elective nature of the procedure, it was frequently cancelled to preserve medical resources during the height of the pandemic.

In addition to the immunological explanation for the increase in mastoidectomies, changes in population behavior may also play an additional role. Thus, there is the possibility of increased social behavior after the end of “social distancing”, which also may have contributed to a rise in the incidence of acute otitis in the population.

The collected data indicates that NPIs have a protective effect against upper and lower respiratory tract infections and their complications, such as acute otitis media. However, due to the increased incidence of complicated otitis media subsequent to the discontinuation of NPIs, they seem unsuitable as sole prophylaxis. Until the year 2022, mastoidectomies were performed relatively rarely. Given the sudden rise in the number of mastoidectomies performed, it is recommended that more ENT surgeons receive training in this specific type of surgery to prevent bottlenecks in patient care.

This study captures the epidemiology of complicated otitis media in the context of the COVID-19 pandemic.

However, there are limitations to the study. The anonymous data collection by procedure code makes it impossible to disaggregate and compare the different indications for mastoidectomy. For instance, the data collected at the local study site shows a higher proportion of mastoidectomies in adults after the COVID-19 pandemic compared to the data from the health insurance companies. This could be based on a relatively low threshold and, therefore, frequent indication for mastoidectomy, e.g., in acute otitis media with inner ear involvement. In addition, there are no available data on the long-term functional outcomes after the mastoidectomies performed. Finally, the study does not review of the incidence of uncomplicated otitis media during the study period. The question remains whether there was an increase in the incidence of more complicated middle ear infections, or if there was an increase in the severity of the disease without a change in incidence of acute otitis media.

## Conclusion

The NPIs introduced during the COVID-19 pandemic had a positive effect on the incidence of other infectious diseases and secondary diseases, such as acute otitis media and its complications, in addition to containing the spread of COVID-19. However, there seems to be a risk of rebound effects due to the reduced immunocompetence of the population, in addition to the negative impact on mental health. Due to the increasing frequency of the usually seldom performed mastoidectomies, further training of ENT-surgeons should be discussed.

## Data Availability

Data are, however, available from the authors upon request and with permission from the regarding health insurance companies.

## Abbreviations

NPI: Nonpharmaceutical intervention
ENT: Ear–nose–throat

## References

[1] Hu T, Podmore B, Barnett R, Beier D, Galetzka W, Qizilbash N, Haeckl D, Weaver J, Boellinger T, Mihm S, Petigara T (2022) Incidence of acute otitis media in children < 16 years old in Germany during 2014–2019. BMC Pediatr. 10.1186/s12887-022-03270-w 35418046 10.1186/s12887-022-03270-w PMC9006409

[2] Otters HB, van der Wouden JC, Schellevis FG, van Suijlekom-Smit LW, Koes BW (2004) Trends in prescribing antibiotics for children in Dutch general practice. J Antimicrob Chemother 53(2):361–366. 10.1093/jac/dkh062 14729760 10.1093/jac/dkh062

[3] Leichtle A, Hoffmann TK, Wigand MC (2018) Otitis media: definition, pathogenesis, clinical presentation, diagnosis and therapy. Laryngorhinootologie 97(7):497–508. 10.1055/s-0044-101327. (**Epub 2018 Jul 9. Erratum in: Laryngorhinootologie. 2018 Jul;97(7):E2**) 29986368 10.1055/s-0044-101327

[4] Loh R, Phua M, Shaw CL (2018) Management of paediatric acute mastoiditis: systematic review. J Laryngol Otol 132(2):96–104. 10.1017/S0022215117001840. (**Epub 2017 Sep 7**) 28879826 10.1017/S0022215117001840

[5] Bakhos D, Trijolet JP, Morinière S, Pondaven S, Al Zahrani M, Lescanne E (2011) Conservative management of acute mastoiditis in children. Arch Otolaryngol Head Neck Surg 137(4):346–350. 10.1001/archoto.2011.29 21502472 10.1001/archoto.2011.29

[6] Brueggemann AB, Jansen van Rensburg MJ, Shaw D, McCarthy ND, Jolley KA, Maiden MCJ, van der Linden MPG, Amin-Chowdhury Z, Bennett DE, Borrow R, Brandileone MC, Broughton K, Campbell R, Cao B, Casanova C, Choi EH, Chu YW, Clark SA, Claus H, Coelho J, Corcoran M, Cottrell S, Cunney RJ, Dalby T, Davies H, de Gouveia L, Deghmane AE, Demczuk W, Desmet S, Drew RJ, du Plessis M, Erlendsdottir H, Fry NK, Fuursted K, Gray SJ, Henriques-Normark B, Hale T, Hilty M, Hoffmann S, Humphreys H, Ip M, Jacobsson S, Johnston J, Kozakova J, Kristinsson KG, Krizova P, Kuch A, Ladhani SN, Lâm TT, Lebedova V, Lindholm L, Litt DJ, Martin I, Martiny D, Mattheus W, McElligott M, Meehan M, Meiring S, Mölling P, Morfeldt E, Morgan J, Mulhall RM, Muñoz-Almagro C, Murdoch DR, Murphy J, Musilek M, Mzabi A, Perez-Argüello A, Perrin M, Perry M, Redin A, Roberts R, Roberts M, Rokney A, Ron M, Scott KJ, Sheppard CL, Siira L, Skoczyńska A, Sloan M, Slotved HC, Smith AJ, Song JY, Taha MK, Toropainen M, Tsang D, Vainio A, van Sorge NM, Varon E, Vlach J, Vogel U, Vohrnova S, von Gottberg A, Zanella RC, Zhou F (2021) Changes in the incidence of invasive disease due to *Streptococcus pneumoniae*, *Haemophilus influenzae*, and *Neisseria meningitidis* during the COVID-19 pandemic in 26 countries and territories in the Invasive Respiratory Infection Surveillance Initiative: a prospective analysis of surveillance data. Lancet Digit Health 3(6):e360–e370. 10.1016/S2589-7500(21)00077-7. (**Erratum in: Lancet Digit Health. 2021 May 26**) 34045002 10.1016/S2589-7500(21)00077-7 PMC8166576

[7] Stamm P, Sagoschen I, Weise K, Plachter B, Münzel T, Gori T, Vosseler M (2021) Influenza and RSV incidence during COVID-19 pandemic-an observational study from in-hospital point-of-care testing. Med Microbiol Immunol 210(5–6):277–282. 10.1007/s00430-021-00720-7. (**Epub 2021 Oct 4**) 34604931 10.1007/s00430-021-00720-7 PMC8487758

[8] Kim SY, Yoo DM, Kim JH, Kwon MJ, Kim JH, Chung J, Choi HG (2022) Changes in otorhinolaryngologic disease incidences before and during the COVID-19 pandemic in Korea. Int J Environ Res Public Health 19(20):13083. 10.3390/ijerph192013083 36293687 10.3390/ijerph192013083 PMC9602729

[9] Windfuhr JP, Günster C (2022) Impact of the COVID-pandemic on the incidence of tonsil surgery and sore throat in Germany. Eur Arch Otorhinolaryngol 279(8):4157–4166. 10.1007/s00405-022-07308-8. (**Epub 2022 Feb 26**) 35218385 10.1007/s00405-022-07308-8 PMC8881894

[10] Buchholz U, Buda S, Lehfeld, A, Loenenbach A, Prahm K, Preuß U, Streib V, Haas W. RKI—GrippeWeb-Wochenbericht KW 30 Kalenderwoche 30 (24.7.–30.7.2023)

[11] Achangwa C, Park H, Ryu S, Lee MS (2022) Collateral impact of public health and social measures on respiratory virus activity during the COVID-19 pandemic 2020–2021. Viruses 14(5):1071. 10.3390/v14051071 35632810 10.3390/v14051071 PMC9146684

[12] De Francesco MA, Pollara C, Gargiulo F, Giacomelli M, Caruso A (2021) Circulation of respiratory viruses in hospitalized adults before and during the COVID-19 pandemic in Brescia, Italy: a retrospective study. Int J Environ Res Public Health 18(18):9525. 10.3390/ijerph18189525 34574450 10.3390/ijerph18189525 PMC8469422

[13] Hullegie S, Schilder AGM, Marchisio P, de Sévaux JLH, van der Velden AW, van de Pol AC, Boeijen JA, Platteel TN, Torretta S, Damoiseaux RAMJ, Venekamp RP (2021) A strong decline in the incidence of childhood otitis media during the COVID-19 pandemic in the Netherlands. Front Cell Infect Microbiol 11:768377. 10.3389/fcimb.2021.768377 34790591 10.3389/fcimb.2021.768377 PMC8591181

[14] Feldman I, Natsheh A, Nesher G, Breuer GS (2022) Social distancing and bacteraemia in the time of COVID-19. Intern Med J 52(2):223–227. 10.1111/imj.15560 34617387 10.1111/imj.15560 PMC8652679

[15] Santarsiero A, Giustini M, Quadrini F, D’Alessandro D, Fara GM (2021) Effectiveness of face masks for the population. Ann Ig 33(4):347–359. 10.7416/ai.2020.2390. (**Epub 2020 Dec 3**) 33258868 10.7416/ai.2020.2390

[16] Cohen R, Ashman M, Taha MK, Varon E, Angoulvant F, Levy C, Rybak A, Ouldali N, Guiso N, Grimprel E (2021) Pediatric Infectious Disease Group (GPIP) position paper on the immune debt of the COVID-19 pandemic in childhood, how can we fill the immunity gap? Infect Dis Now 51(5):418–423. 10.1016/j.idnow.2021.05.004. (**Epub 2021 May 12**) 33991720 10.1016/j.idnow.2021.05.004 PMC8114587

[17] Oh KB, Doherty TM, Vetter V, Bonanni P (2022) Lifting non-pharmaceutical interventions following the COVID-19 pandemic—the quiet before the storm? Expert Rev Vaccines 21(11):1541–1553. 10.1080/14760584.2022.2117693. (**Epub 2022 Sep 5**) 36039786 10.1080/14760584.2022.2117693

[18] Viñeta Paramo M, Ngo LPL, Abu-Raya B, Reicherz F, Xu RY, Bone JN, Srigley JA, Solimano A, Goldfarb DM, Skowronski DM, Lavoie PM (2023) Respiratory syncytial virus epidemiology and clinical severity before and during the COVID-19 pandemic in British Columbia, Canada: a retrospective observational study. Lancet Reg Health Am 30(25):100582. 10.1016/j.lana.2023.100582 10.1016/j.lana.2023.100582 PMC10495630 37705884

[19] Çağlar HT, Pekcan S, Yılmaz Aİ, Ünal G, Ercan F, Savaş S, Akcan ÖM, Ünsaçar MZ, Ünsaçar K, Özdemir M (2023) The epidemiologic trend of respiratory syncytial virus has returned strongly to its origin after the pandemic: five-year data from a single center. Pediatr Pulmonol. 10.1002/ppul.26696 37737535 10.1002/ppul.26696

[20] Kiefer A, Pemmerl S, Kabesch M, Ambrosch A (2023) Comparative analysis of RSV-related hospitalisations in children and adults over a 7 year-period before, during and after the COVID-19 pandemic. J Clin Virol 166:105530. 10.1016/j.jcv.2023.105530. (**Epub 2023 Jul 6**) 37481874 10.1016/j.jcv.2023.105530

[21] Alert from the French Health Ministry, December 6th 2022, DGS-URGENT N°2022_83

